# Supplementary material for: Antibacterial Activity of Propolis-Embedded Zeolite Nanocomposites for Implant Application
Source: Materials (Basel). 2021 Mar 3;14(5):1193. doi: 10.3390/ma14051193 (PMC7961988; doi:10.3390/ma14051193)
Supplement: Supplementary file 1 [file materials-14-01193-s001.pdf]

materials

Supporting information

Antibacterial Activity of Propolis-Embedded Zeolite Nanocomposites for Implant Application

Jun Sik Son [1], Eun Ju Hwang [2], Lee Seong Kwon [3], Yong-Gook Ahn [2], Byung-Kwon Moon [2], Jin Kim [4], Douk Hoon Kim [5], Su Gwan Kim [6,*], Sook Young Lee [2,7,*]

[1] IT-Bio Material Research Team, Korea Textile Development Institute, Daegu 41842, Korea; sonjk1@empas.com
[2] Smart Medical Convergence Technology Support Center, Chosun University, Gwangju 61012, Korea; ejlife0827@naver.com (E.J.H.); ygookahn@naver.com (Y.-G.A.); mbkbusy@gmail.com (B.-K.M.)
[3] RAPHA BIO Co. Ltd., Wanju-Gun, Jeollabuk-do 55367, Korea; eskwon1946@hanmail.net
[4] Dental Healthcare & Clinical Trial Center, Chosun University, Gwangju 61452, Korea; cream4251@hanmail.net
[5] Research Center, Medical Division, Nexturn Co. Ltd., Gyeonggi-do 17086, Korea; doukhoon@naver.com
[6] Sangmu Su Dental Clinic, Gwangju 61998, Korea
[7] Regional Innovation Center for Dental Science & Engineering, Chosun University, Gwangju 61452, Korea
* Correspondence: seedbank2001@hanmail.net (S.-Y.L.); sugwankim@daum.net (S.G.K.); Tel.: +82-629-737-662 (S.Y.L.); Fax: +82-629-737-662 (S.Y.L.)

Results

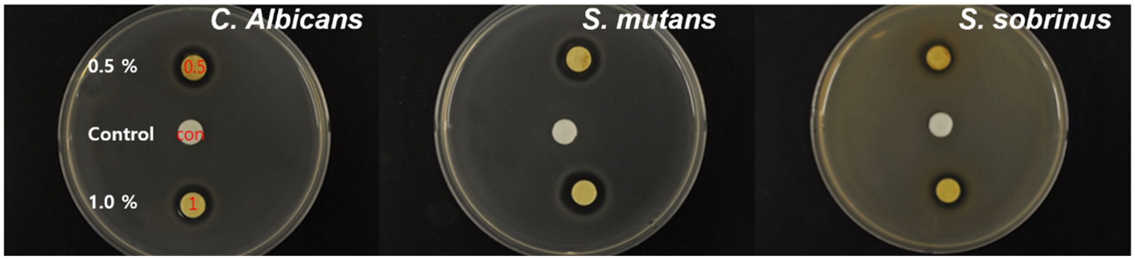

Figure S1. Antibacterial activity of propolis.

Table S1. Antibacterial activity of propolis.

	Inhibition zone (Dia, mm)		
	C. *albicans*	S. *mutans*	S. *sobrinus*
Control	-	-	-
0.5%	5.0 mm	6.0 mm	4.0 mm
1%	5.0	6.5	4.0

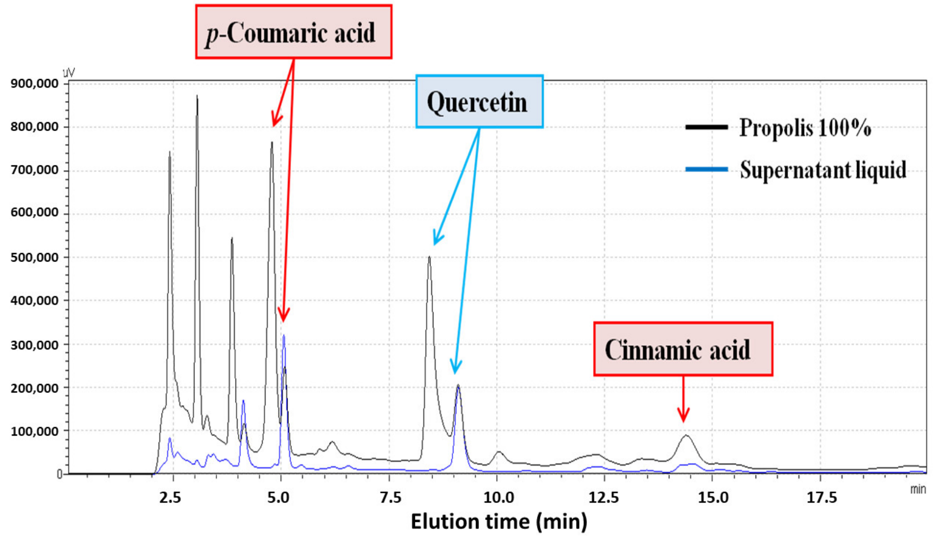

Figure S2. HPLC of propolis before (blue line) and after (black line) embedding into nanocomposutes.

Table S2. Condition of compounding Propolis-embedded zeolite/PLA-PCL pellets of Figure 3.

Zone	C1	C2	C3	C4	C5	C6	C7	C8	C9	Adapter	Die
Temperature (°C)	120	135	135	140	145	145	150	150	160	160	160
Melt pressure = 1 kg/cm³						Temperature = 161 °C					
Main motor = 15 amp						Exturder-speed = 99.5 r/min					
Screw feeder = 10 rpm											

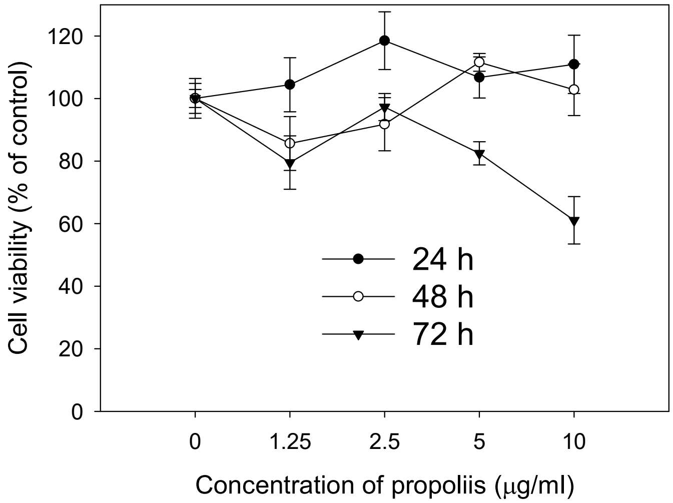

Figure S3. The effect of propolis on the viability of normal (HaKaT) cells.
